# High expression of PD-L1 on conventional dendritic cells in tumour-draining lymph nodes is associated with poor prognosis in oral cancer

**DOI:** 10.1007/s00262-024-03754-x

**Published:** 2024-07-02

**Authors:** Hjalmarsson Eric, Krzysztof Piersiala, Vilma Lagebro, Pedro Farrajota Neves Da Silva, Marianne Petro, Magnus Starkhammar, Alexandra Elliot, Rusana Bark, Gregori Margolin, Susanna Kumlien Georén, Lars-Olaf Cardell

**Affiliations:** 1 Division of ENT Diseases, Department of Clinical Sciences, Intervention and Technology, Karolinska Institutet, Stockholm, Sweden; 2 Department of Otorhinolaryngology, Karolinska University Hospital, Stockholm, Sweden; 3 Department of Pathology and Cytology, Karolinska University Hospital, Stockholm, Sweden; 4 Department of Head and Neck Surgery, Medical Unit Head Neck Lung and Skin Cancer, Karolinska University Hospital, Stockholm, Sweden

**Keywords:** Oral squamous cell carcinoma, Dendritic cells, T-cells, Tumour draining lymph node, Flow cytometry, PD-L1

## Abstract

**Introduction:**

Oral squamous cell carcinoma (OSCC), while common and with a favorable prognosis in early stages, presents a marked reduction in survival rate upon metastasis to lymph nodes. Early detection of lymph node metastasis via biomarkers could enhance the therapeutic strategy for OSCC. Here, we explored dendritic cells (DCs) and cytotoxic T-cells in tumour-draining lymph nodes (TDLNs) as potential biomarkers.

**Method:**

Dendritic cells and cytotoxic T-cells in 33 lymph nodes were analyzed with multi-parameter flow cytometry in TDLNs, regional non-TDLNs surgically excised from 12 OSCC patients, and compared to 9 lymph nodes from patients with benign conditions.

**Results:**

Our results displayed a higher proportion of conventional cDC1s with immunosuppressive features in TDLN. Further, high PD-L1 expression on cDC1 in TDLNs was associated with metastasis and/or recurrent disease risk. Also, elevated levels of memory CD8^+^ T-cells and terminally exhausted PD-1^+^TCF-1^−^CD8^+^ T-cells were observed in TDLNs and non-TDLNs compared to healthy lymph nodes.

**Conclusions:**

We conclude that TDLNs contain cells that could trigger an anti-tumor adaptive response, as evidenced by activated cDC1s and progenitor-like TCF-1^+^ T-cells. The detection of high PDL1 expression on cDC1s was indicative of TDLN metastasis and an adverse prognosis, proposing that PD-L1 on dendritic cells in TDLN could serve as a predictive biomarker of OSCC patients with a worse prognosis.

**Supplementary Information:**

The online version contains supplementary material available at 10.1007/s00262-024-03754-x.

## Introduction

Oral squamous cell carcinoma (OSCC) remains one of the most prevalent malignancies of the head and neck region [1]. It constitutes a significant global health concern due to its incidence and mortality rates [2]. The prognosis is generally favorable in its early, localized stages, with a relatively high 5-year survival rate. However, this optimistic outlook is overshadowed when the disease metastasizes to nearby lymph nodes, drastically reducing the chances of survival [3]. This stark contrast in patient outcomes underscores the necessity of developing improved strategies for enhanced detection of patients with poor prognoses. In combination with tumor staging, immunological biomarkers could further improve the detection of high-risk OSCC patients, enabling customized treatment protocols and the initiation of novel therapies.

The therapeutic landscape for OSCC has undergone significant advancements over recent decades. Innovations in surgical procedures advances in radiation therapy, and the development of new chemotherapeutic agents have collectively contributed to this progress [4]. The introduction of immune checkpoint inhibitors (ICIs) targeting programmed cell death protein 1 (PD-1) and its ligand (PD-L1) has revolutionized the treatment of several solid tumors, leading to notable improvements in patient survival rates [5]. However, these improvements have been modest in the treatment of OSCC with only approximately 20% of OSCC patients experiencing a favorable response to the treatment [6, 7]. Biomarkers have been tested to stratify patients that would benefit from ICIs and the most common biomarker used in the treatment of OSCC has been PD-L1 expression on tumor cells [8]. The complexity of the mechanism behind ICIs is highlighted by the observation that some patients with PD-L1 negative tumors still respond to therapies targeting this pathway [9, 10]. Conversely, this indicates a more intricate process than the mere local disruption of PD-1 activation within the tumor. Furthermore, immunotherapy has been recently shown to have a higher efficacy in head and neck cancer when used in the neoadjuvant setting [11–14]. One of the possible explanations for this phenomenon, confirmed in animal studies, is that before surgery, tumor-draining lymph nodes (TDLNs) are usually intact and can provide a robust anti-tumor immune response induced by immunotherapy [15].

Dendritic cells (DCs) have a well-established and critical role in anti-tumor immunity [16]. Through the uptake of tumor antigens and presenting them to CD8^+^ T-cells, they foster the formation of specific cytotoxic T-cells, with the ability to specifically target and eradicate cancer cells [17]. This highlights the adaptive immune system’s capacity to recognize and aggressively combat tumor growth. Human DCs are typically categorized into conventional Type 1 (cDC1), conventional Type 2 (cDC2), and plasmacytoid (pDCs) [18]. cDC1 are characterized by low expression of CD123, CD11c, moderate expression of CD1c and high expression of CD141. cDC2 are characterized by low expression of CD123, and moderate expression of CD141, CD11c and CD1c. pDC are characterized by low expression of CD11c, CD1c, CD141 and high expression of CD123 [19]. For inciting a potent CD8^+^ T-cell response, cDC1s are deemed crucial due to their exceptional capacity to activate CD8^+^ T-cells and facilitate Th1 polarization of CD4^+^ T-cells [20, 21]. Although cDC2s and pDC also activate CD8^+^ T-cells, their functions are predominantly associated with the activation of various CD4^+^ Th subtypes and modulation of DC function [22–25].

T-cell-mediated tumor immunity efficacy relies on the migration of DCs to the TDLNs and their subsequent maturation, enabling T-cell activation [26]. Activated T-cells then egress from the lymphatics and migrate to the primary tumor. Within the tumor microenvironment (TME), the expression of PD-L1 on both tumor and immune cells acts as a regulatory checkpoint, driving the exhaustion of activated CD8^+^ T-cells [27]. In a study by Wang et.al they discovered that CD8^+^ Tex^term^ T-cells (TCF-1^−^PD1^+^) and CD8^+^ T-cells Tex^prog^ T-cells (TCF1^+^PD1^+^) are the dominating CD8^+^ T-cell populations within he TME, and that a high fraction of CD8^+^ Tex^term^ T-cells promote survival [28].

Although the interplay between DCs and CD8^+^ T-cells within TDLNs is essential for anti-tumor immunity and appears critical for the response to ICI therapy, prior research has predominantly focused on tumor immunity within the primary tumor site and peripheral blood [29]. We have refined surgical techniques to address this gap to enable the precise identification and sequential analysis of TDLNs and regional non-draining lymph nodes in OSCC [30, 31]. This advancement facilitates a more in-depth investigation of the locoregional immune landscape and its impact on therapeutic outcomes.

This study aims to explore the immunological underpinnings of OSCC, focusing on the role of dendritic cells and exhausted CD8^+^ T-cells in TDLN and their possible use in patient prognosis.

## Method

### Patient characteristics

Eligible patients enrolled in this study met the following inclusion criteria: (1) diagnosis of OSCC in the floor of the mouth or tongue. (2) Willingness to participate in the study. Exclusion criteria were as follows: (1) systemic autoimmune diseases (2) synchronous second malignancies, hemo-lymphopoietic malignancies in the past (3) any other acute or chronic condition that could influence the immunological milieu in lymph nodes. All of the enrolled subjects were staged as cN0 preoperatively. Human Papillomavirus (HPV) stands out as a significant risk factor for HNSCC, particularly in oropharyngeal cancers. However, the role of HPV in the carcinogenesis of OSCC remains debatable. Prior studies have indicated a low prevalence of HPV-positivity in OSCC tumors. A recent meta-analysis, encompassing data from over 5000 patients across 24 countries, revealed that the estimated prevalence of HPV-positive OSCC stands at 6% (95% CI; 3–10%) [32]. Many of the studies included in the meta-analysis used HPV-DNA as a method to confirm HPV positivity. However, the presence of HPV-DNA positivity does not necessarily indicate biological activity or direct involvement in cancer development. Given the generally low prevalence and limitations inherent in numerous studies examining HPV positivity in OSCC, the patients studied within this project are considered to be HPV-negative. The study cohort's follow-up time ranged from 17 to 50 months, with an average of 33.7 months. For other clinical patient characteristics, see Table 1 Patient characteristics.

**Table 1:** Clinical and pathological data on enrolled patients

Patient no.	Location of the tumour	pT-stage	pN-stage	M-stage	Recurrence (Yes/No)
1	Tongue	T1	N0	M0	No
2	Floor of the mouth	T2	N0	M0	No
3	Tongue	T3	N1	M0	No
4	Tongue	T3	N0	M0	No
5	Floor of the mouth	T2	N0(i +)	M0	No
6	Floor of the mouth	T1	N0	M0	No
7	Tongue	T1	N0	M0	Yes
8	Tongue	T3	N0	M0	Yes
9	Tongue	T3	N0	M0	No
10	Tongue	T2	N0	M0	No
11	Tongue	T3	N0	M0	No
12	Tongue	T1	N0	M0	No

Twelve patients diagnosed with oral squamous cell carcinoma (OSCC) and treated through primary surgery involving sentinel node identification and removal were included in the study. Each patient’s specimen comprised one sentinel node (TDLN) and one non-sentinel node (n-TDLN), both subjected to analysis. The mean age of the cohort was 68 years, ranging from 46 to 85 years. Among the participants, seven were female (58%), and five were male (42%). Tumors were primarily located in the tongue (67%) and remaining in the floor of the mouth (33%). Regarding pathological tumor staging, three subjects were classified as pT1 (25%), three as pT2 (25%), and six as pT3 (50%). Of the twelve patients, 83% (10 patients) exhibited no metastasis in their TDLNs, while 17% (2 patients) had a positive nodal status. All subjects exhibited no distant metastasis (M0). Notably, two cases experienced recurrence, while the remaining ten did not. For Clinical and pathological data on enrolled patients, see Table 1.

Additionally, nine lymph nodes were obtained from individuals that underwent surgery for benign conditions, specifically benign parotid or submandibular gland lesions. These lymph nodes, collected with ethical approval and informed consent are referred to as healthy lymph nodes (HLNs) in the context of this study.

### Sample retrieval

Patients enrolled in the study went through a TDLN detection procedure at Karolinska University Hospital, Stockholm, Sweden, the method has been described in our previous work [33]. The method is further described in Supplementary Material.

### Sample preparation

Cryopreserved single-cell suspensions from human lymph nodes were thawed as described in Supplementary methods. For the analysis of DCs, 3*10^6^ cells were incubated with Human Fc-block (BD Biosciences, New Jersey, USA #564,220) and then with the following antibodies in the dark for 20 min: anti-CD40 (5C3), anti-HLA-DR (G46-6), anti-CTLA4 (BNI-3), anti-CD123 (9F5), anti-CCR7 (2-L1-A),anti-CD33 (WM-53), anti-CD86 (BU63), anti-CD141 (1A4), anti-CD1c (F10/21A3), anti-PD-L1 (MIH4), anti-PD-1 (EH12.1), anti-CD80 (L207.4), anti-CD11c (B-ly6), anti-CD45 (Hi30), anti-CD3 (SK7), anti-CD19 (HIB19), anti-CD20 (2H7) and anti-CD14 (MφP9), (BD Biosciences, New Jersey, USA) see Supplementary Table S1 for more details. Stained cells were washed and resuspended in a Fixable Far Red Dead Cell Stain Kit (Invitrogen, Massachusetts, USA, #L34974) and incubated in the dark for 20 min at room temperature. Prepared cells were then washed and fixed in 1% paraformaldehyde. For the analysis of T cells, 0.5*10^6^ cells were incubated with the following antibodies in the dark for 20 min: anti-CD4 (RPA-T4), anti-CCR7 (2-L1-A), anti-CD8 (HIT8a), anti-PD-1 (EH12.1) and anti-CD3 (SP34-2), (BD Biosciences, New Jersey, USA), see Supplementary table S2 for more details. Stained cells were washed and resuspended in a Fixable Far Red Dead Cell Stain Kit and incubated in the dark for 20 min at room temperature. A transcription buffer KIT (BD Biosciences, New Jersey, USA, #562574) was used according to the manufacturer’s instructions for the intra-nuclear staining of anti-TCF-1 (S33-966) or isotype control (MOPC-31C) (BD Biosciences, New Jersey, USA) was used, see Supplementary Table S2 for more details. Prepared samples were acquired on an LSR Fortessa with FACSdiva software 6.0 (BD Biosciences, New Jersey, USA). Generated 3.0 FCS files were analyzed with FlowJo software (10.9.0).

### t-SNE and FlowSOM

First, seven samples from TDLN, n-TDLN, and HLN were imported into the FlowJo software for phenotype characterization. DCs were gated as described in Supplementary Fig. S1. Next, the gated DC population was exported to generate a new file, including only the DC population. Then, the samples in the new file were individually normalized to 3895 cells using the downsample plugin (v.3.3.1). The samples were subsequently exported and put together using the concatenation tool in FlowJo. A t-SNE was performed to reduce the dimensions of the multiparameter data. The following parameters were used to create the t-SNE: iterations = 1000, perplexity = 30, learning rate (eta) = 2224, KNN algorithm: exact (vantage point tree), and gradient algorithm: Barnes–Hut. Clusters of phenotypically related cells were then detected by the FlowJo plugin FlowSOM (v.3.0.18) [34]. FlowSOM was run with the following parameters: CD141, CD11c, CD45, CD123, CD33, CD1c, number of meta clusters = 6, SOM grid size 10 × 10, plot channels all as pie charts, node scale 100%, background meta clusters, generate heatmap. To characterize the DC expression of receptors and ligands, samples from the five patients with cDC1 PD-L1^High^, paired n-TDLN, and five patients with cDC1 PD-L1^Low^ were imported to FlowJo. Next, the cDC population was exported into a new file. To maximize resolution, four samples in all three compartments were normalized to 2476, and one sample (all three compartments) was normalized to 688 cells resulting in equal number of cells in each compartment. The parameters analyzed were HLA-DR, CD80, CD86, PD-L1, PD-1, CTLA-4 and CCR7. FlowSOM number of meta cluster = 3. The same phenotype markers were used in t-SNE and FlowSOM.

### Statistics

Statistical analyses were performed with GraphPad Prism version 9.4.1 (GraphPad Software, La Jolla, CA). The Shapiro–Wilks normality test was used to determine if data sets were normally distributed. For paired data, Wilcoxon matched pair signed rank test or two-tailed paired t-tests were chosen, depending on the distribution of the data. For unpaired data Mann–Whitney rank test or Un-paired two-tailed t-test was used depending in the distribution. For comparison of more than two groups, one-way ANOVA with Holm–Šídák correction was performed for normally distributed data, and Kruskal–Wallis with Dunn´s correction tests were used for comparisons of more than two groups not meeting the assumption of data normality. *P* < 0.05 (*) was considered significant, and *P* < 0.01 (**), *P* < 0.001 (***), *P* < 0.0001 (****) were considered highly significant.

### Ethical statement

All procedures performed in this study involving human participants were approved by the Swedish Ethical Review Authority and followed the ethical standards and guidelines of the institutional and/or national research committee and the Declaration of Helsinki. Informed and signed consent was obtained from all individual participants included in the study. This study involved human participants and was approved by the Ethics Committee (2015/1650–31/2 and 2019–03518.).

## Results

In total 21 individuals of which 12 were diagnosed OSCC patients were included in this study. Samples were taken from three compartments, TDLN, n-TDLN and HLN. The HLNs were taken from individuals undergoing surgery for benign conditions, specifically benign parotid or submandibular gland lesions. The samples were analyzed with flow cytometry using extracellular and intracellular marker panels.

### Enhanced dendritic cell fractions in tumor-draining lymph nodes

**Fig. 1:**
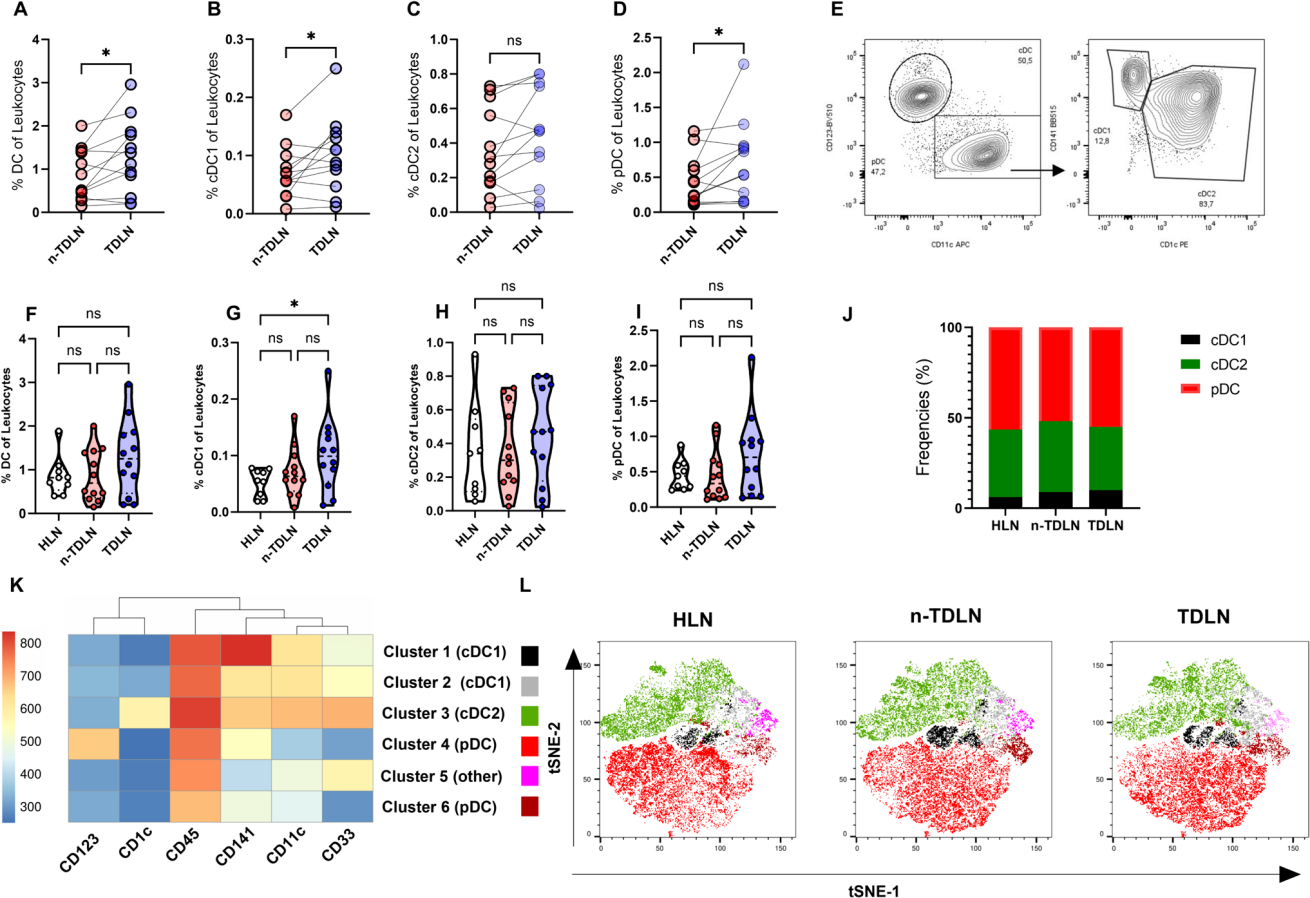
Increased fraction of cDC1 in TDLN. **A**–**D**, **F**–**I** Percentage of DCs, cDC1, cDC2, and pDC of leukocytes. **E** representative flow cytometry dot plot. **J** Stacked bar graph of the relative fraction of DC subtypes. **K** Heatmap of the mean expression of markers used for DC phenotyping. **L** t-SNE visualization of FlowSOM cluster analysis of CD141, CD11c, CD45, CD1123, CD33, CD11c of total DC. Truncated Violine plots display mean and extend between the minimum and maximum value. *p* < 0.05 = *

Flow cytometric analysis with multiple parameters indicated a statistically significant enrichment of DC populations in TDLNs compared to n-TDLNs, with a *p*-value of 0.013 (see Fig. 1A). Subtype analysis showed that cDC1 and pDC were found in larger proportions in TDLNs than in n-TDLNs (*p* = 0.017 and *p* = 0.02, respectively, see Fig. 1B, D). No significant difference could be detected for cDC2, see Fig. 1C. Moreover, TDLNs exhibited a significant increase in the fraction of cDC1 when compared to healthy lymph nodes (HLNs) with a *p*-value of 0.02 (see Fig. 1G). No significant difference was seen between the compartments for cDC, cDC2 and pDC (see Fig. 1F, H–I). No significant differences were observed in the relative proportions of DC subtypes among TDLNs, n-TDLNs, and HLNs (see Fig. 1J). For the gating strategy, see Fig. 1E; a full gating strategy is presented in Supplementary Fig. S1. The data was also analyzed by performing a dimensionality reduction of the multiparameter data using t-distributed Stochastic Neighbor Embedding (t-SNE) and the clustering algorithm FlowSOM. We identified six distinct DC clusters, corroborating the detection uniformity of cDC1, cDC2, and pDC across all lymph node types (Fig. 1K, L). The phenotype of cluster one and two was CD141^++^CD11Cc^+^CD1c^−^CD123^−^ and CD11c and corresponded with cDC1. Cluster 3 had the phenotype CD141^+^CD11c^+^CD1c^+^CD123^−^ which is consistent with cDC2. The phenotype for cluster four and six was CD141^+^CD11c^−^CD1c^−^CD123^+^wich is consistent with pDC. Cluster five expressed CD141^−^CD11c^+^CD1c^−^CD123^−^ and did not align with the phenotypes of known cDC or pDC populations. Detailed expression intensities for each cluster have been compiled in Supplementary Fig. S2.

### Elevated expression of co-stimulatory molecules and PD-L1 in cDC1 of TDLNs

**Fig. 2:**
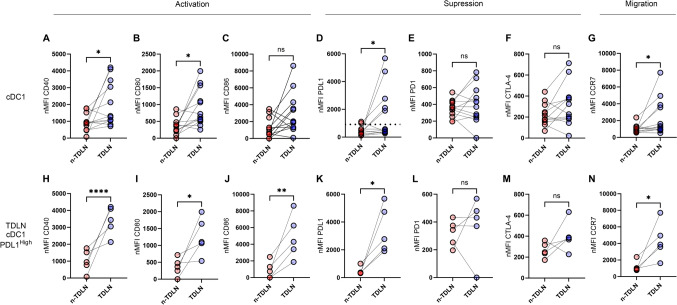
Increased expression of PD-L1 on cDC1 in TDLN. **A**–**G** Display paired expression level data for cDC1, all patients included. **D** Cut-off level for cDC1 PD-L1^high^ and cDC1 PD-L1^low^ in TDLN. **H**–**N** Display paired expression level data for cDC1, selected for patients with cDC1 PD-L1^high^. Truncated Violine plots display mean and extend between the minimum and maximum value. *p* < 0.05 = *. *p* < 0.01 = **, *p* < 0.0001 = ****

Using multi-parametric flow cytometry, we examined the expression of membrane proteins critical for the induction of T-cell responses by dendritic cells, focusing on co-stimulatory, suppressive, and migratory molecules. In TDLNs, cDC1 exhibited a significant upregulation of the co-stimulatory molecules CD40 and CD80 compared to their counterparts in n-TDLNs, with *p*-values of 0.03 and 0.01, respectively (Fig. 2A, B). Moreover, cDC1 in TDLNs showed enhanced expression of the inhibitory ligand PD-L1 (*p* = 0.048; Fig. 2D), a key modulator of immune response. No appreciable changes were observed in the levels of CD86, PD-1, or CTLA-4 (Fig. 2C, E and F respectively). The analysis was extended to CCR7, a receptor instrumental in DC migration, which was found to be expressed at higher levels in cDC1 of TDLNs compared to n-TDLNs (*p* = 0.046; Fig. 2G).

Notably, a subset of patients (5 out of 12) demonstrated a distinctly high expression of PD-L1 in their TDLNs (Fig. 2D). We established a mean fluorescence intensity (MFI) threshold to categorize patients into a cDC1 PD-L1^High^ and PD-L1^Low^ group. We determined the cut-off to be 914, the maximum MFI value in the PD-L1^Low^ group (575) plus three standard deviations (338,75). The cut-off is shown in Fig. 2D as a dotted line.

The patients that were detected as cDC1 PD-L1^high^ were gated and the three cDC1, cDC2 and pDC phenotypes were compared between TDLN and n-TDLN. Our findings indicate that the cDC1 PD-L1^high^ patient group had a higher expression of PD-L1, co-stimulatory and migratory molecules in TDLNs when compared to n-TDLNs, see Fig. 2H–K, N. This pattern was not observed in the cDC1 PD-L1^low^ group, see Supplementary Fig. S3. In the cDC1 PD-L1^high^ patient group subsequent evaluation of cDC2 mirrored the results seen in cDC1, with an increase in CD40, CD80, PD-L1, and CCR7 expression in TDLNs detailed in Supplementary Fig. S4. In contrast, the plasmacytoid dendritic cells (pDCs) showed no differential expression between TDLNs and n-TDLN, (data not shown).

### Enhancement of immunosuppressive dendritic cells in cDC1 PD-L1^High^ patient group

**Fig. 3:**
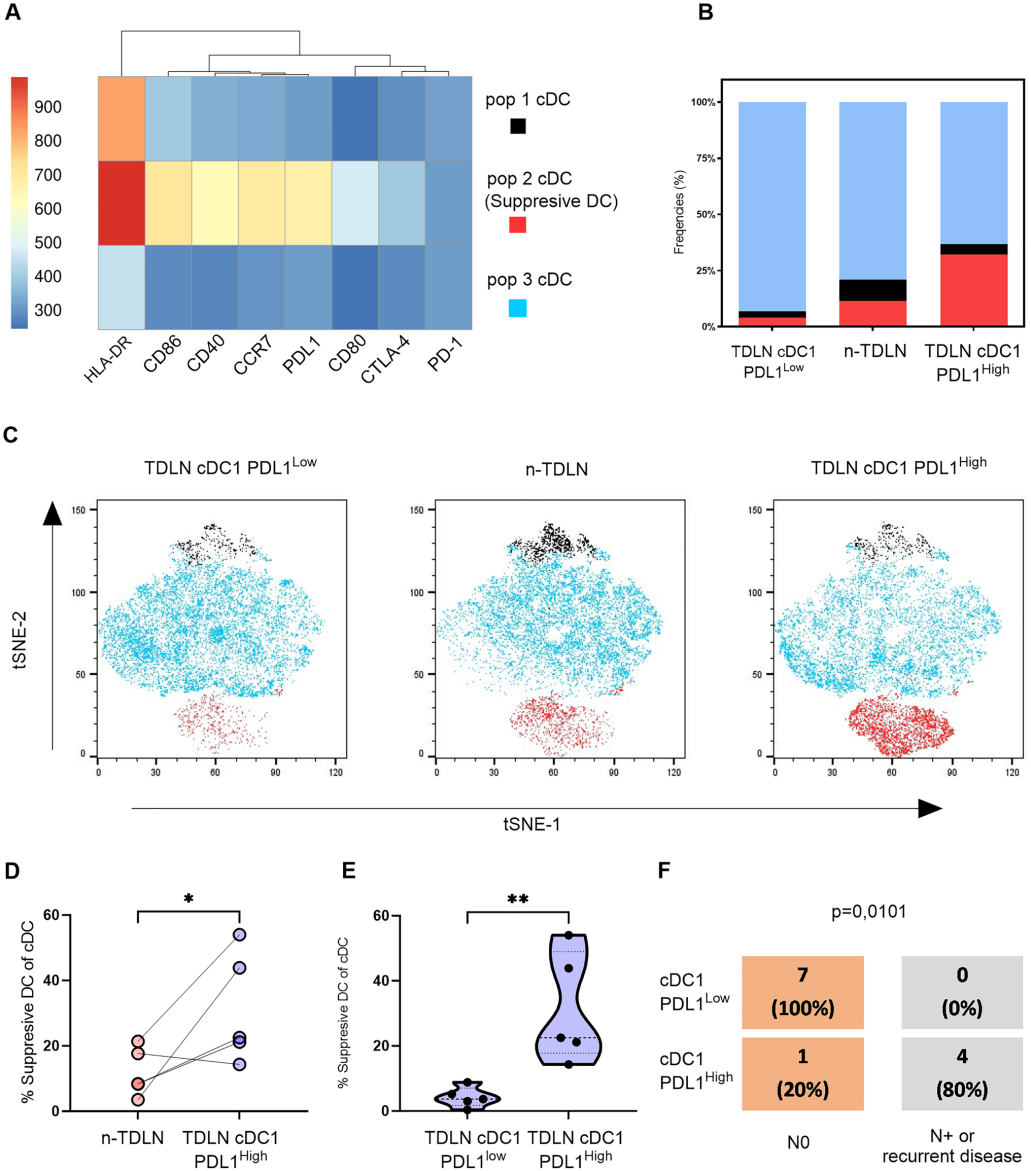
Suppresive DCs is inceased in TDLN of patients categorized as cDC1 PD-L1High. A tSNE and FlowSOM algorithm was conducted on the cDC1 PD-L1High group with their paired n-TDLNs and the cDC1 PD-L1Low group. **A** Heatmap illustrating three clusters with their mean expression of the markers HLA-DR, CD86, CD40, CCR7, PDL1, CD80, CTLA-4, and PD-1. **B** Stacked bar graph of the relative fraction of DC subtypes. **C** Analysis visualizing the different cDC clusters. **D**–**E** Percentage of Suppresive cDCS (cluster 2) of cDCs, **F** Fisher’s exact test displaying the association between cDC1 PFL1^High^ and the increased risk of nodal involvement. Truncated Violine plots display mean and extend between the minimum and maximum value. *p* < 0.05 = *. *p* < 0.01 = **

We analyzed the DC activation profiles among the patient groups, cDC1 PD-L1^High^ and the cDC1 PD-L1^Low^ group and compared TDLN and n-TDLN. The expression on n-TDLN in the cDC1 PD-L1^Low^ group did not show any activation in the earlier analyses and was therefore excluded from this analysis. The dimensionality reduction analysis employing t-distributed Stochastic Neighbor Embedding (tSNE) and the clustering algorithm FlowSOM was performed, with the incorporation of the markers CD40, CD80, CD86, PD-L1, PD-1, and CTLA-4 on the DC population. This analytical approach revealed three distinct DC clusters which were represented in all patients, see Fig. 3A–C. Cluster 1 was characterized by high HLA-DR expression; Cluster 2 showed a robust expression of HLA-DR, CD40, CD86, CCR7, PD-L1, and CTLA-4, a profile indicative of immunosuppression; and Cluster 3 exhibited low expression across all measured parameters, see Fig. 3A.

Our analysis indicated that Cluster 2 was significantly more prevalent in the cDC1 PD-L1^High^ group compared to both paired n-TDLNs and TDLNs from the cDC1 PD-L1^Low^ group (*p*-values: 0.047 and 0.008 respectively), see Fig. 3D and E. Further investigation of disease manifestation into lymph nodes and disease relapse revealed that within the cDC1 PD-L1^Low^ group, eight patients were classified as N0, with no instances of disease relapse noted during the follow-up period. In contrast, the cDC1 PD-L1^High^ group consisted of five patients, of which two initially staged as N0 experienced recurrence post-surgery, and two presented with cancer cells detected in the TDLN at the time of surgery, see Table 1. Fisher’s exact test indicated an elevated risk of nodal involvement for patients categorized as cDC1 PD-L1^High^, see Fig. 3F.

### Elevated levels of exhausted CD8 + T-cells in regional lymph nodes

**Fig. 4:**
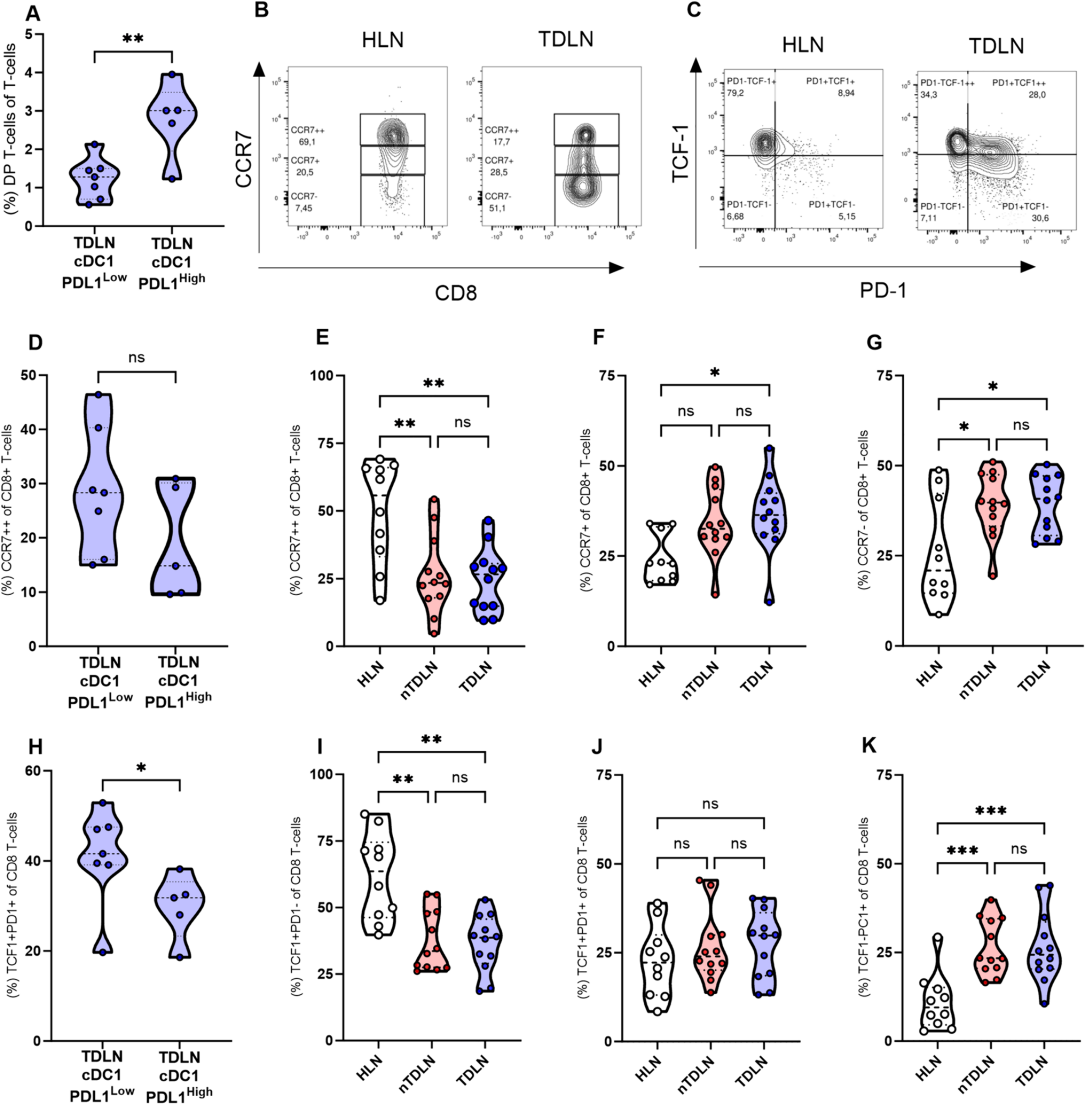
Patients categorized as cDC1 PD-L1High display an increased fraction of exhausted CD8^+^ and DP T-cells in TDLN. **A** Frequency of double positive (DP) T-cells. **B**, **C** representative flow cytometry dot plot. **D**–**G** Frequency of naïve (CCR7^++^), central memory (CCR7^+^), and effector memory (CCR7-) in CD8 T-cells. **H**–**K** Frequency of non-exhausted (TCF-1^+^PD-1^−^), exhausted (TCF^−^1^+^PD-1^+^), and terminally exhausted (TCF-1^−^PD-1^+^) in CD8^+^ T-cells. Truncated Violine plots display mean and extend between min. and max. < 0.05 = *, *p* < 0.001, *p* < 0.001 = ***

Flow cytometric analysis was employed to assess CD8^+^ T-cell memory differentiation in TDLN, n-TDLN, and HLN. We began by dividing patients into two groups based on cDC1 expression of PD-L1, one group with high expression of PD-L1 and one with low expression of PD-L1. In TDLN, we detected an increased fraction of double-positive T-cells (DP T-cells) in the PD-L1 high group (*p* = 0.0055; Fig. 4A). This difference was not detected in n-TDLN; see Supplementary Fig. S5. No difference could be detected in the CD4/CD8 ratio (*p* = 0.8763; Supplementary Fig. S6) or in the fraction of naïve CD8^+^ T-cells between the PD-L1 high and low group in TDLN (*p* = 0.2677; Fig. 4D). We then quantified naïve (CCR7^++^), central memory (CCR7^+^), and effector memory (CCR7^−^) CD8^+^ T-cell in HLN, n-TDLN, and TDLN indicated by the expression of CCR7 (Fig. 4B). Our findings revealed a decreased proportion of naïve T-cells in both TDLN and n-TDLN when compared to HLN (*p* = 0.0014 and *p* = 0.0019, respectively; Fig. 4E). Conversely, an enrichment of memory T-cell fractions was observed. Specifically, TDLNs exhibited a significantly increased proportion of both central and effector memory T-cells compared to HLNs (*p* = 0.016 and *p* = 0.0179; Fig. 4F, G), while n-TDLNs only showed an enrichment in the effector memory subset (*p* = 0.0179; Fig. 4G).

Subsequent evaluation of T-cell exhaustion, indicated by the expression of TCF-1 and PD-1 (Fig. 4C), demonstrated a substantial reduction in the fraction of non-exhausted T-cells (TCF-1^+^PD-1^−^) in both TDLN and n-TDLN as compared to HLN (*p* = 0.007 and *p* = 0.003, respectively; Fig. 4F). No difference in the fraction of CD8^+^ Tex^prog^ T-cells could be detected comparing HLN, n-TDLN and n-TDLN, see Fig. 4G. In contrast, the proportion of CD8^+^ Tex^term^ T-cells (TCF-1^−^PD-1^+^) was significantly higher in both TDLN and n-TDLN compared to HLN (*p* = 0.007 for both; Fig. 4H). When dividing patients into cDC1 PD-L1 high and low we could detect an increased fraction of non-exhausted cells in the PD-L1 low group in TDLN (*p* = 0.0303; Fig. 4E). This difference was not detected in n-TDLN (Supplementary Fig. S5).

## Discussion

Taking advantage of our unique access to unfixed samples, this investigation has highlighted the crucial role of immune activation within TDLNs of patients with OSCC. To the best of our knowledge, it is the first study using flow cytometry to investigate phenotypes of dendritic cells and CD8^+^ T cells in TDLNs and n-TDLNs of patients with OSCC and healthy lymph nodes from the head and neck.

We observed that cDC1s in TDLNs exhibit significantly elevated immune checkpoint molecule PD-L1 expression. The heightened expression of PD-L1 on cDC1 in TDLN was detected in 5 of 12 patients, these patients also displayed an increased fraction of cDC with features of immune suppression and were associated with an increased risk of metastasis of TDLN or disease recurrence. The data also reveals a prevalent state of CD8^+^ T cell exhaustion across n-TDLN and TDLN, which may underpin the impaired immune response often seen in OSCC. Importantly, n-TDLN and TDLNs were found to house cDC1, and TCF-1^+^PD-1^+^ stem cell-like T-cells, suggesting a retained capacity to mount an anti-tumor adaptive response.

In the TDLN of OSCC patients particularly, cDC1s and pDCs subsets were increased. cDC1s, are known to have the ability to trigger de novo T cell responses after migrating to TDLNs and drive CD8^+^ T-cell responses to immunotherapy with checkpoint inhibitors [35–37]. Shenkel et al. found that the number of cDC1s in TDLNs decreases with tumor progression and that their presence and activation in TDLNs is crucial for sustaining CD8^+^ T cell responses in the tumor [38]. Therefore, our finding displaying TDLN as the primary target for cDC1s migrating from the tumor in patients with head and neck cancer could explain why there is a higher response rate to immune checkpoint inhibitors in the neoadjuvant setting as opposed to adjuvant administration. In the later scenario, the primary surgical treatment has typically removed TDLNs rich in cDC1s, leading to reduced response rates.

To the best of our knowledge, our study is the first to show increased levels of pDC in TDLNs’. Our data is in line with data from melanoma patients, where pDC is shown to be increased in TDLN, and were demonstrated to correlate with poor prognosis [39, 40]. It has previously been shown that pDC in HNSCC is dysfunctional, with reduced capability to support T-cell mediated immunity and may increase PD-L1 during disease progress [41, 42]. This highlights that pDC may have important roles both for sustaining and disrupting anti-tumor immunity in OSCC patients.

In our study, the cDC1s in TDLNs were further characterized by an increased expression of co-stimulatory molecules (CD40 and CD80) and CCR7 compared to n-TDLNs. This finding suggests that cDCs in TDLNs in OSCC are functional and capable of effectively inducing T-cell responses. In contrast to our finding, van Pul et al. demonstrated that cDC1s show a significant reduction in CD40, CD83, and CD86 expression in pre-metastatic TDLNs in breast cancers [29]. It is known that both anti-inflammatory (IL-6, IL-10, IDO, MGSF and transforming growth factor β1) and pro-inflammatory (IFN-α, IL-2, IL-15, IL21) cytokines are released within the TME, and that this greatly influences maturation of DCs that migrate to TDLN [43]. It is possible that OSCC TME are more pro-inflammatory compared to breast cancer affecting maturation differently.

As previously described, dendritic cells may in response to an anti-inflammatory environment adopt a regulatory or immunosuppressive program characterized by expressing immune checkpoint molecules such as PD-L1 [36, 44]. These immunosuppressive phenotypes can impair the priming of CD8^+^ T cells in TDLNs and promote tumor immune evasion and progression [36, 44]. In our cohort of 12 patients, we identified five characterized by high expression of PD-L1 on cDC1s in TDLNs. These patients also displayed an increased fraction of cDC with features of immune suppression like PD-L1 and CTLA-4. Interestingly, two out of five patients with PD-L1^high^ DCs had metastasis in the analyzed TDLN. The remaining two patients, who had no metastasis detected by a pathologist in the examined nodes, developed tumor recurrence after surgical treatment. Our analysis confirmed a higher risk of metastasis or recurrent disease in this selected patient group. Thus, it is not unconscionable to think that immunosuppressive cDC1s disrupt T-cell immunity in the advanced stages of the disease, promoting the escaped immune system surveillance. Our findings align with previous animal studies, which showed that cDC1s express more PD-L1 than n-TDLNs from the same animal [15]. Furthermore, the expression of PD-L1 on DCs was proven essential for responsiveness to PD-L1 blockade in a mouse model [36].

To further understand the capacity of TDLNs to produce an anti-tumor adaptive immune response, we investigated CD8^+^ T cells in HLN, n-TDLN, and TDLN. Compared to HLN, n-TDLN, and TDLN exhibited increased memory cell differentiation and signs of exhaustion, indicating that T-cell activation and expansion of T-cell mediated anti-tumor immunity is not restricted to TDLN alone. While TDLNs are considered the primary source of tumor-reactive T-cells and targets for immune therapeutic interventions [45]. n-TDLN also represents an opportunity for local cancer treatment. In TDLN, dendritic cells activating tumor-specific T-cells have been shown to be subjected to tumor-derived factors like IL-6, TGF-β, prostaglandin-E2, and VEGF [46]. Our study demonstrated that immune suppressive cDC1s could be identified in TDLN in a fraction of the patients using flow cytometry. These cDC1s were associated with increased CD8^+^ T-cell exhaustion and poor prognosis making them a significant target for prognostic evaluation and for future treatment interventions. Additionally, we observed an increase in DP T-cells in TDLN among patients categorized with immunosuppressive cDC1s. Earlier studies have described elevated levels of DP T-cells in autoimmune diseases, viral infections, and cancers. In melanoma, increased levels of DP T-cells in lymph nodes have been associated with metastasis and tumor cell infiltration [47]. This aligns with our data, suggesting that the increase in DP T-cells in TDLNs may be linked to local immune suppression and the formation of a metastatic niche.

While our study has provided valuable insights into the biology of DCs TDLN, it is crucial to acknowledge the limitations that may affect the interpretation of our findings. First and foremost, the sample size for our investigation was modest, comprising 12 cancer patients. However, we have clearly demonstrated the presence of immunosuppressive DCs in TDLN and the association of cDC1 with high expression of PD-L1 with increased CD8^+^ T-cell exhaustion in TDLN and increased risk of metastasis or recurrent disease. Additionally, our project was limited to flow cytometric analysis of dissociated samples. As a result, we are not able to describe the immune suppressive function of cDC1 beyond the expression of PD-L1. These challenges present opportunities for refinement in future investigations.

Our findings provide new insights into the immune potential of TDLNs in patients with OSCC. Animal studies have shown that TDLNs are crucial for mediating the response to immune checkpoint blockade and serve as an essential source of new T cells ascending into the tumor during immunotherapy [15]. Preclinical models investigating the administration of immune checkpoint inhibitors directly into TDLNs demonstrated that this approach drives effective tumor control and improves the response to immune checkpoint blockade compared to systemic administration [48]. The identification of high PD-L1 expression on cDC1s in TDLNs as a predictor of metastasis or recurrent disease highlights its potential as a valuable prognostic biomarker. Detecting this immunosuppressive phenotype in TDLNs could enable early stratification of patients with a poor prognosis, aiding in personalized treatment planning and potentially improving patient outcomes. Our data underscore the significance of TDLNs and n-TDLNs in generating a robust anti-tumor response. Administering ICIs in the neoadjuvant setting before surgical resection of the primary tumor might maximize therapeutic efficacy by leveraging the intact TDLNs to elicit a strong immune response. Given that the immune potential of TDLNs plays a crucial role in shaping the response to checkpoint blockade, administering ICIs directly into TDLNs could be a possible approach to bolster anti-tumor immunity. Such localized treatments might enhance response rates and minimize systemic toxicity, offering a tailored and effective therapeutic option for OSCC patients. The association of high PD-L1 expression on cDC1s with increased T-cell exhaustion and poor prognosis suggests that targeting these immunosuppressive dendritic cells could be a promising therapeutic strategy. Therapeutic interventions aimed at reducing PD-L1 expression or reversing immunosuppressive features on cDC1s might restore T-cell functionality, enhancing the effectiveness of immunotherapies. Combining ICIs with treatments that specifically modulate the dendritic cell function in TDLNs could be beneficial. For instance, agents that promote cDC1 maturation or block inhibitory molecules like PD-L1 might synergize with ICIs to provide more comprehensive anti-tumor immunity.

In summary, our study elucidates the immunological dynamics within TDLNs in OSCC and points toward promising clinical implications. Future research should focus on developing tailored therapeutic strategies that exploit the immune potential of TDLNs, ultimately improving outcomes for patients with oral cancer.

### Supplementary Information

Below is the link to the electronic supplementary material.

Supplementary file1 (DOCX 1979 KB)

## Data Availability

Data is provided within the manuscript file or within the supplementary file.
