# Supplementary material for: High expression of PD-L1 on conventional dendritic cells in tumour-draining lymph nodes is associated with poor prognosis in oral cancer
Source: Cancer Immunol Immunother. 2024 Jul 2;73(9):165. doi: 10.1007/s00262-024-03754-x (PMC11219651; doi:10.1007/s00262-024-03754-x)
Supplement: Supplementary file 1 — Supplementary file1 (DOCX 1979 KB) [file 262_2024_3754_MOESM1_ESM.docx]

**Supplementary Methods**

*Tissue dissociation*

After surgical excision, unfixed tissue samples were kept in pre-chilled MACS Tissue Storage Solution (Miltenyi Biotec, Bergisch Gladbach, Germany, #130-100-008). A Tumor Dissociation Kit (Miltenyi Biotec, Bergisch Gladbach, Germany, #130-095-929) was used to dissociate the samples enzymatically and mechanically. After dissociation, cells were filtered through a 100 µm Cell Strainer (Corning, Glendale, AZ, USA #352360) and viable frozen as described in the supplementary methods.

*Cryo preserve cells*

1. Use DMEM + 10% FBS and DMEM + 10%FBS + 20% DMSO
2. Dilute cells in 500µl DMEM + 10% FBS per cryo tube, add dropwise DMEM + 10%FBS + 20% DMSO and doble the volume.
3. Transfer 1ml cell suspension onto a cryo tube.
4. Freeze cells in mr frosty -80, and for long term storage at -180.

*Thaw cells*

1. Warm cryo tube in water bath at 40^o^c until almost thawed.
2. Transfer cell suspension into a 15ml falcon tube with 10ml warm media (DMEM + 5% FBS).
3. Wash cells two times with PBS, 300g, 5min.

*Sample retrieval*

The clinical routine for sentinel lymph node biopsy (SLNB) at Karolinska University Hospital, Stockholm, Sweden is briefly performed as follows. Approximately 16 hours before surgery, patients receive an injection of a radiotracer (99mTc-tilmanocept or 99mTc-nanocolloid). The radiotracer is injected under the mucosa in the submucous space around the tumor (in total 0.5-0.6 ml), which is equal to 70-80 MBq. A SPECT/CT is performed the following morning shortly prior to surgery to detect the TDLN A handheld gamma probe (Neoprobe®, Gamma Detection System, Mammotome-Vingmed) detects the TDLN in the operative field during surgery. A fluorescent indocyanine green dye (ICG) is injected peritumorally at the start of the surgery, and a handheld imager (SPY-PHI, Stryker) is used to confirm the location of the TDLN. After surgery, the TDLNs are examined histopathologically by a pathologist.

*Flow cytometry Instrument setup and compensation*Purified lymphocytes were used to adjust the instrument voltage to 2.5 x rSDen. FACS Diva application settings were then used to reduce PMT variability over time; for the daily tracking of the LSR Fortessa, cytometer set-up and tracking beads (BD Biosciences, New Jersey, USA) were used daily before sample acquisition. Compensation was performed in FACSdiva Software with single stained anti-mouse beads (BD Biosciences, New Jersey, USA), anti-rat beads (BD Biosciences, New Jersey, USA), and ArC reactive beads (Thermo Scientific, Massachusetts, USA, #A10346).

**Supplementary Tables**

Table S1. Antibody panel for flow cytometry


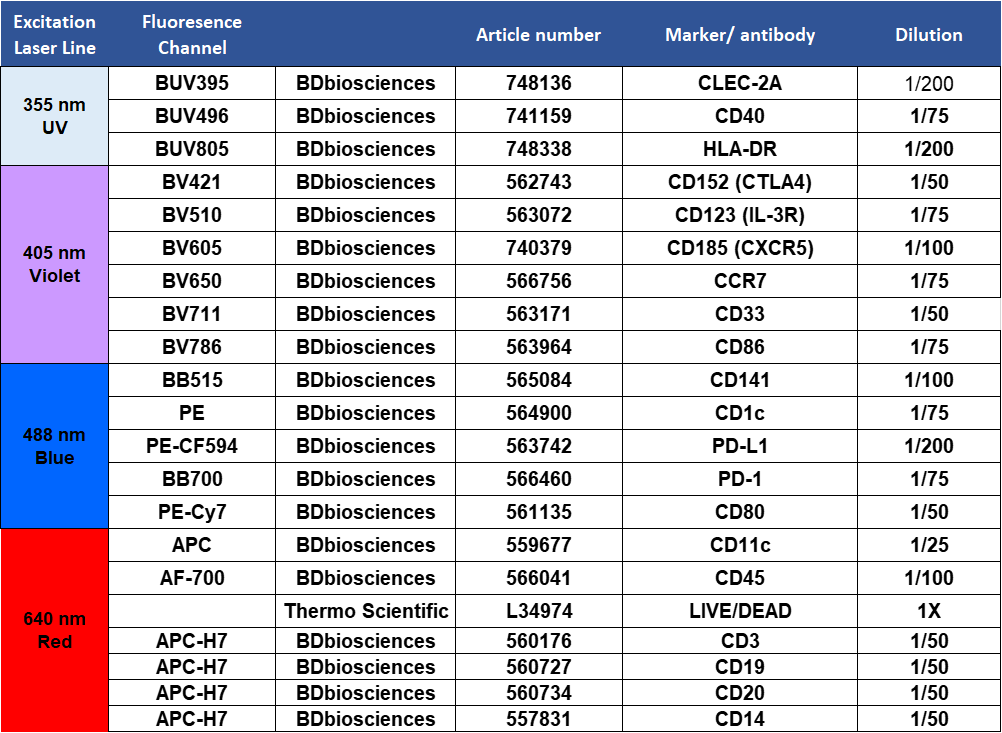


Table S2. Antibody panel for flow cytometry analysis of T cells.


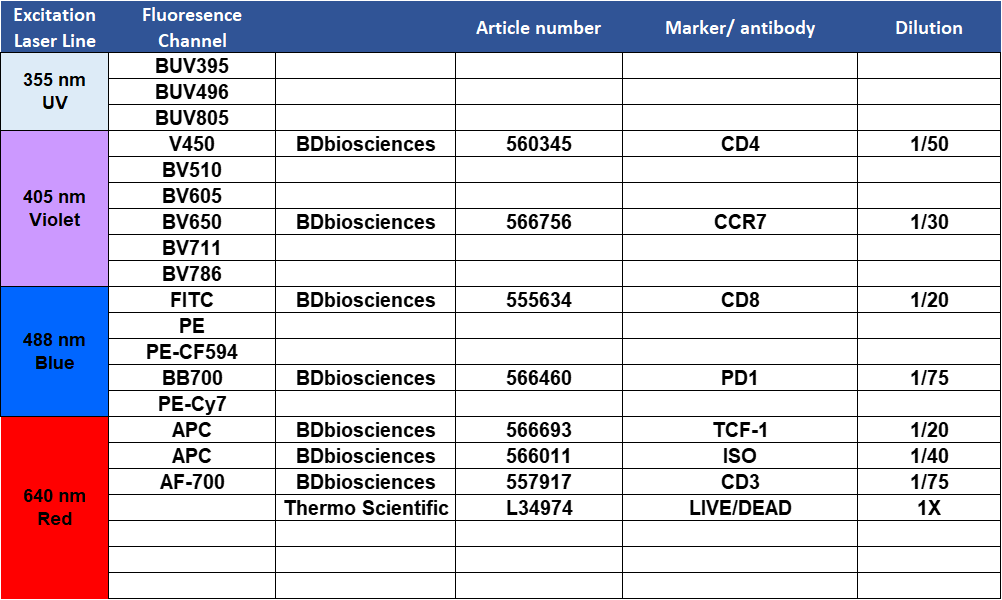


**Supplementary Figures**

Figure S1. Flow cytometry gating strategy of the DC population.

**
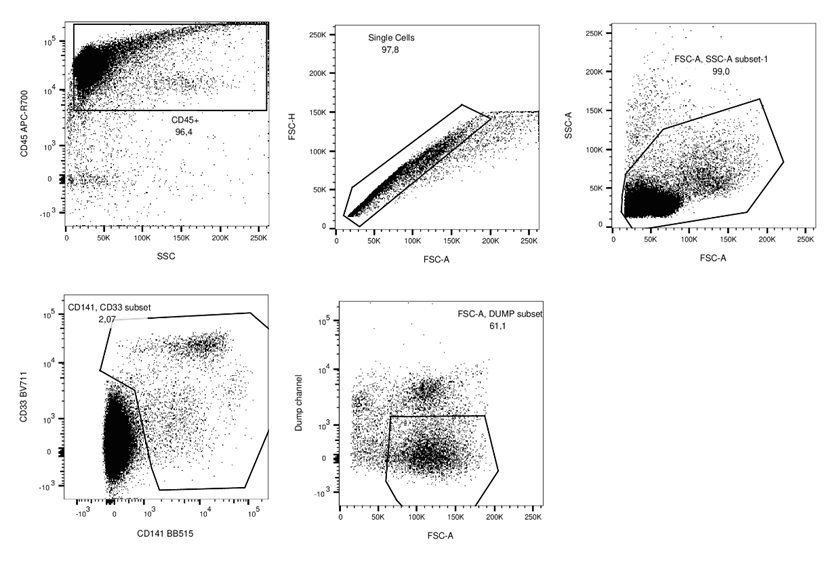
**

Figure S2. Expression intensities for each cluster.


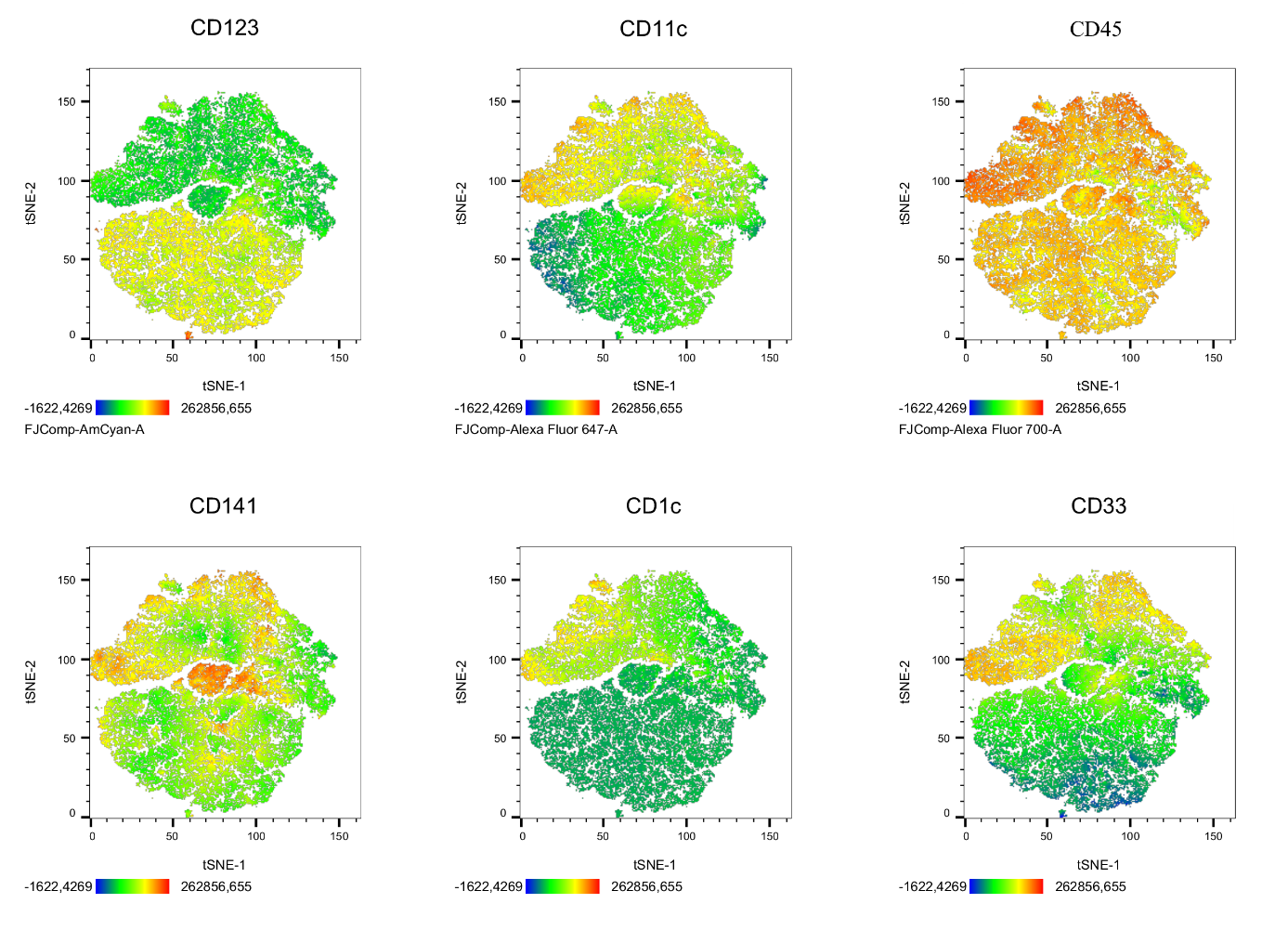


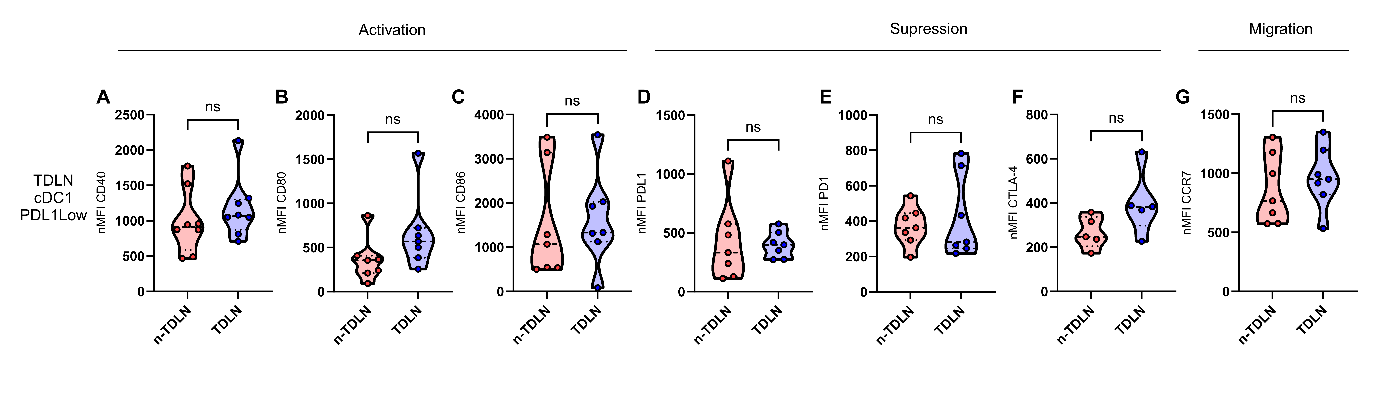


Figure S3. No differential expression of co-stimulatory and migratory molecules in TDLNs compared to n-TDLNs was observed in the cDC1 PD-L1^Low^ group.


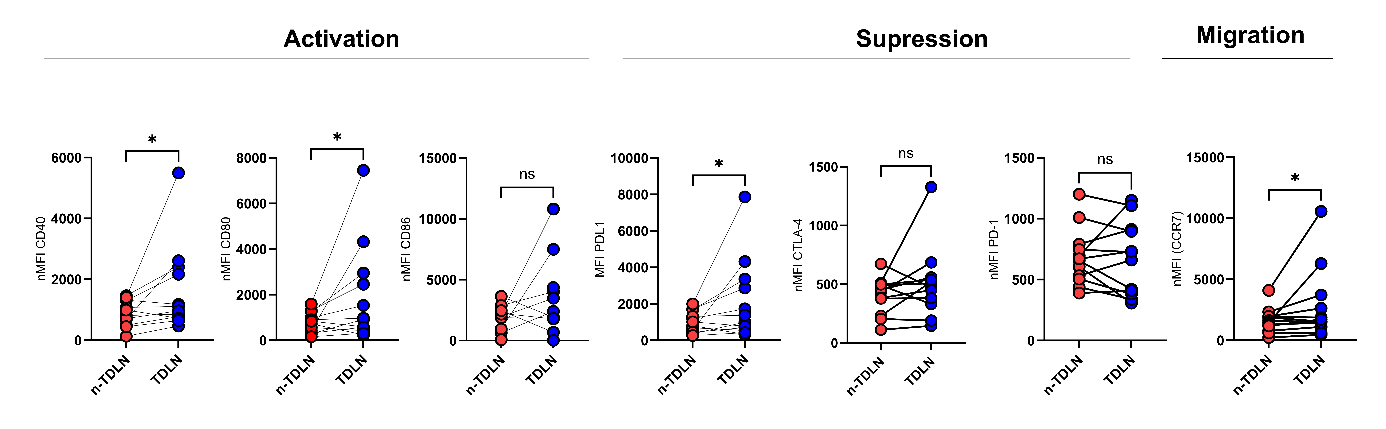


Figure S4. Evaluation of cDC2 showed an increase in CD40, CD80, PD-L1, and CCR7 expression in TDLNs compared to n-TDLN.


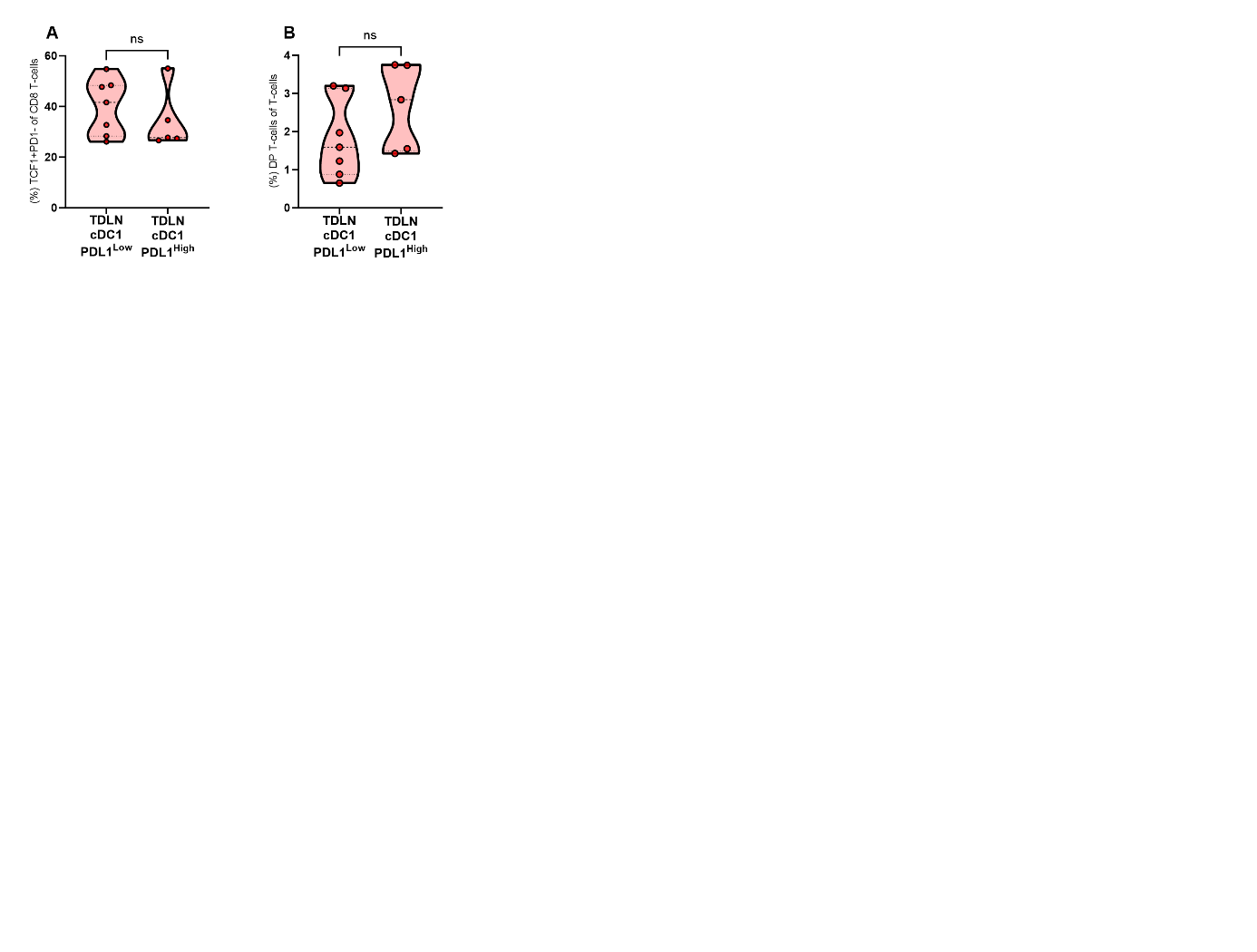


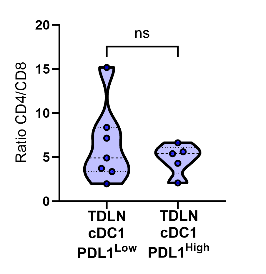
Figure S5. Evaluation of TCF-1^+^PD1^-^ T-cells and DP-T-cells in n-TDLN in patients categorized based on PDL-1 expression of cDC1 in TDLN. DP= double-positive T-cells.

Figure S6. Evaluation of CD4/CD8 T-cell ratio in TDLN in patients categorized based on PDL-1 expression of cDC1 in TDLN.
